# Unveiling social relationships: exploring the importance of relationships as a moderator of the link between effort-reward imbalance and leader-member exchange among healthcare professionals

**DOI:** 10.1186/s12889-024-19652-x

**Published:** 2024-08-10

**Authors:** Rebecca Erschens, Ines Armbruster, Sophia Helen Adam, Felicitas Rapp, Lisa Braun, Carla Schröpel, Stephan Zipfel, Monika A. Rieger, Harald Gündel, Eva Rothermund, Florian Junne

**Affiliations:** 1 Department of Psychosomatic Medicine and Psychotherapy, University Hospital Tuebingen, University of Tuebingen, 72076 Tuebingen, Baden-Wuerttemberg Germany; 2 Department of Anesthesiology and Intensive Care Medicine, University Hospital Ulm, Ulm University, 89070 Ulm, Baden-Württemberg Germany; 3 German Center for Mental Health (DZPG), Tübingen, Germany; 4 Institute of Occupational and Social Medicine and Health Services Research, University Hospital Tübingen, 72076 Tuebingen, Baden-Wuerttemberg Germany; 5 Department of Psychosomatic Medicine and Psychotherapy, Ulm University Medical Center, 89081 Ulm, Baden-Württemberg Germany; 6 Department of Psychosomatic Medicine and Psychotherapy, Otto von Guericke University Magdeburg, University Hospital Magdeburg, 39120 Magdeburg, Sachsen-Anhalt Germany

## Abstract

**Objective:**

Healthcare professionals are at increased risk of experiencing occupational stress and its detrimental stress-sequalae. Relevant theories that contribute to the subjective experience of occupational stress have been identified, such as the model of effort-reward imbalance (ERI) and the concept of leader-member exchange (LMX). The aim of this study was to examine how the perceived importance of social relationships at work moderates the relationship between LMX and imbalance ERI.

**Methods:**

A survey was conducted among *N* = 1,137 healthcare professionals from diverse occupational categories in a tertiary hospital in Germany. ERI was gauged using the German version of the Effort-Reward Imbalance Questionnaire (ERI-S 10). The quality of leader-employee dyadic relationships was assessed using the German version of the Leader-Member Exchange (LMX-7). The importance of social relationships was assessed on the basis of a previously validated polarity profile.

**Results:**

More than 75% of healthcare professionals reported high levels of ERI, with those involved in direct patient care particularly affected. On average, leaders rated relationship quality higher than their respective followers. Subjectively higher LMX was associated with lower ERI. This association was moderated by the perceived importance of social relationships at work. Higher subjective ratings of their importance led to a stronger association.

**Conclusion:**

The study highlights the particular challenges faced in the healthcare sector. The results emphasize that the perceived importance of social relationships at work can play a key role in healthcare professionals’ job stress and underline the need for stress prevention programs that engage both leaders and followers.

## Introduction

The modern workplace within healthcare organizations such as hospitals is diverse and challenging as a wide range of healthcare professionals such as doctors, nurses, administrators, clinical services work together with the common goal of providing exemplary patient care. This task unfolds amidst increasing challenges such as streamlined work processes, shortages of skilled personnel, global crises and pandemics, demographic shifts, and limited financial resources [1–4]. Therefore, the interaction of these factors can lead to suboptimal working conditions, increasing the potential for raised absenteeism and economic loss, compromised patient care, as well as having a lasting negative impact on the wellbeing of healthcare professionals [5–8]. Previous research has highlighted the prevalence of emotional and psychological distress and workload burden among healthcare professionals [9–12].

Meta-analytic evidence underlines the high risk of stress-related disorders among healthcare workers. Approximately 25% of nurses struggle with increased emotional exhaustion, approximately 22% experience decreased personal accomplishment, and approximately 15% report increased symptoms of depersonalization [13]. Similarly, Kunzler et al. [14] highlight an increased susceptibility to depression, post-traumatic stress disorder, and even a higher incidence of suicide among healthcare professionals compared to other work areas.

The effort-reward imbalance (ERI) model, pioneered by Siegrist [15], is a cornerstone in the understanding of occupational stress in the context of work-related satisfaction crises (gratification crises). Central to this model is the postulation of a reciprocal interplay between job strain and the subsequent rewards or gratifications obtained [16]. A harmonious balance between the effort expended and rewards received can significantly enhance emotions, general well-being and health [17]. Hence, distress arises when there is an incongruence between the effort of work and the reward experienced. This incongruity, mathematically characterized by an ERI greater than 1, indicates an imbalance [17].

Individuals who report an ERI are three times more likely to experience negative physical health manifestations than those who do not experience such an imbalance [18–20]. The effects of ERI extend to various domains, including reduced occupational well-being, [21–23] increased job dissatisfaction [18, 24], and a spectrum of bio-psycho-social consequences at the behavioral level [25–27]. In health care, the impact of ERI is far-reaching as its level emerges as a predictive factor for nurses’ intention to quit their jobs after one year [28] and is correlated with reduced quality of patient care [29].

In this context, the concept of leader-member exchange (LMX) presents a highly relevant paradigm [30]. LMX, initially developed by Graen et al. [31], is a theoretical approach that describes the quality of the relationship between a leader and an employee. The LMX focuses on the differentiated dyadic and mature relationship between leaders and followers that can be mutually beneficial [30, 31]. The LMX theory suggests that leaders weave unique relational patterns with each follower, resulting in different qualities of LMX for different individuals [32, 33].

In healthcare, empirical evidence highlights that superior LMX quality is intertwined with favorable psychosocial working conditions for followers [34], increased job satisfaction [35], and reduced intentions to leave the current position [36]. There is also a focus on the important role of LMX in the area of stress and health, where robust LMX has a stress-reducing effect and can promote employee well-being [1, 37, 38]. Research on the connection between LMX and ERI have revealed a negative correlation [39]. This suggests that stronger LMX bonds may serve as a protective shield against the hazards of ERI, potentially creating a healthier work environment for all involved. Thus, social support between leader and follower emerges as buffer to dampen the impact of distress and mitigate the potentially detrimental effects of ERI on mental well-being. The rising importance of social support within the healthcare sector is further underscored by its association with positive health behaviors and improved health outcomes, as demonstrated by studies by Jolly et al. [40] and Wang et al. [41]. In a study conducted by Junne et al. [42], healthcare professionals were surveyed to assess the perceived importance of work-related stressors with a polarity profile (for more information see methods section). The study found that one of the most important stressors in the sample was the importance of social relationships at the workplace.

In particular, the provision of social support from colleagues and family acts as a bulwark against occupational burnout among physicians [43]. Conversely, inadequate or poor support from colleagues and supervisors, or interpersonal conflict, can increase vulnerability to mental disorders in the top percentile [44].

**Fig. 1:**
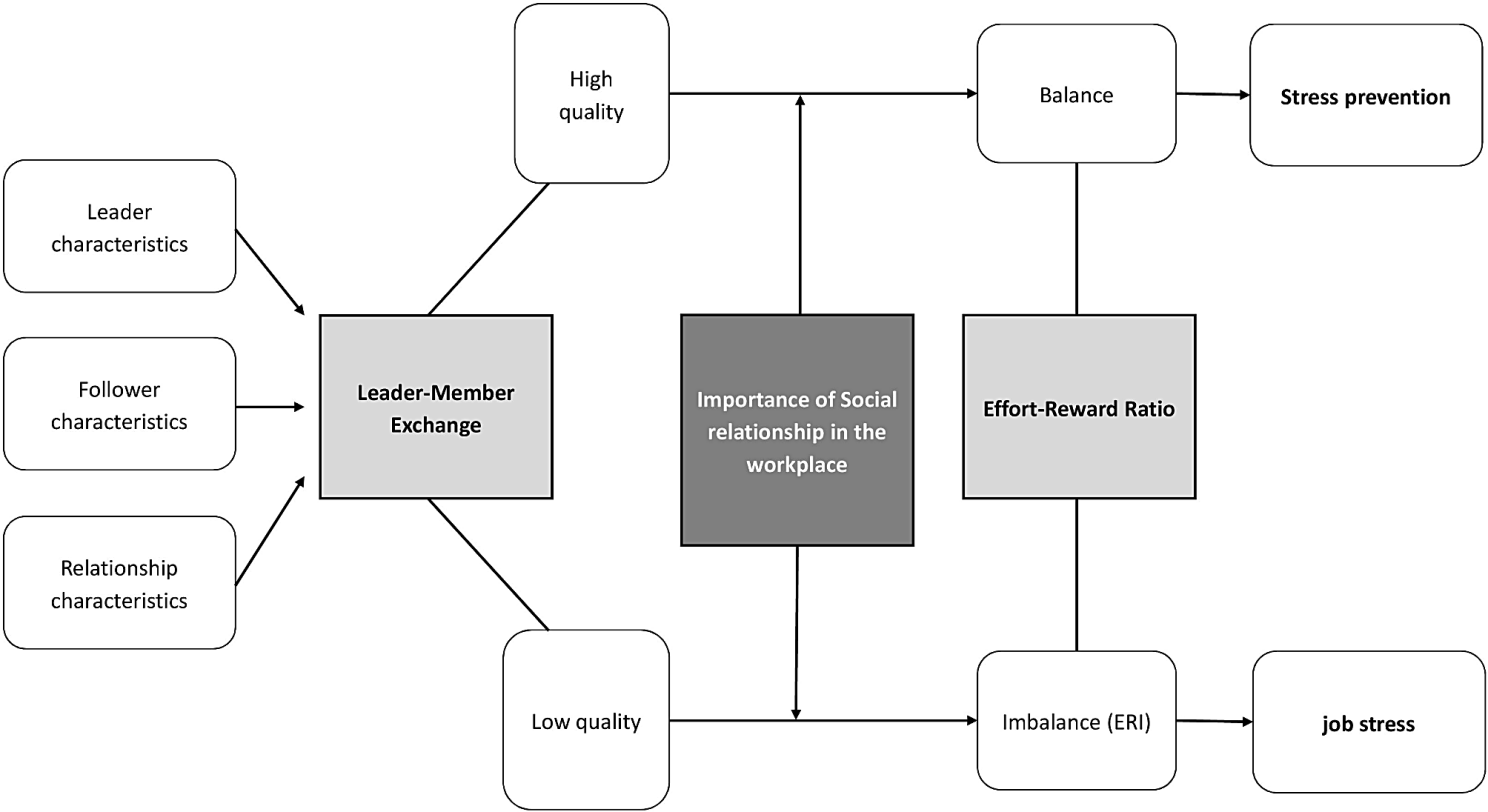
Overview of our conceptual model which is based on findings from the leader-member exchange concept (LMX, [30]) and the effort-reward imbalance model (ERI, [23]), and also includes the potential influence of social relationships in the workplace [42, 45]

Despite the apparent importance of these relationships, the scientific terrain regarding the interplay and mechanisms linking these constructs – ERI and LMX and social relationships - remains relatively unexplored. Thus, our central focus is to examine a potential moderating impact of the perceived importance of social relationships in the workplace on the dynamic interplay between LMX, the quality of LMX and the presence of ERI. Figure 1 illustrates our conceptual model, which is explained in more detail below and is based and adapted from the conceptual framework of LMX (see Graen et al. [30]), and ERI (see Siegrist et al. [23]), including the possible impact of the importance of social relationships (see Junne et al. [42]; Michaelis et al. [45]).

As indicated in the conceptual model, the quality of a leader-follower relationship can be affected by three core factors [32], follower characteristics, and relational characteristics. Leader characteristics include gratification-seeking behaviors, a transformational leadership approach [32] and personality traits such as extraversion or conscientiousness [46]. Follower characteristics may manifest as a strong need for leadership [47], emotional intelligence [48] or job performance [49]. The third aspect pertains to the nature of the relationship itself. For instance, a study by Sin et al. [50] demonstrated that the quality of the LMX relationship is contingent upon the intensity of interactions within the relationship and the duration of the relationship. Another significant factor influencing the quality of the relationship is the degree of similarity between the personal characteristics of the leader and the follower. A greater degree of similarity is associated with a higher LMX [51].

Depending on the quality of the relationship (high vs. low), two trajectories may emerge: Previous studies suggest that low quality leader-member exchange (LMX) is associated with increased stress, leading to negative health outcomes for followers [52, 53]. Conversely, high quality LMX has been shown to be associated with reduced job stress, thereby promoting follower well-being. The literature suggests a negative relationship between LMX and ERI across occupational domains, which may lead to improved leader-follower relationship quality and reduced work-related stress among followers [39].

We hypothesize that the perceived importance of social relationships within the workplace exerts a moderating role on the association between LMX and the ERI ratio. This implies that the relationship between LMX and the ERI ratio may fluctuate depending on the perceived importance of workplace social relationships. Consequently, we infer that this moderating relationship suggests that followers’ beliefs play a central role in shaping how social interactions affect their stress experience.

The present study focuses in particular on the role of the follower. To date, there has been insufficient research into the impact of the perceived importance of social relationships at the workplace on the interplay between the leader-member exchange model and the effort-reward imbalance framework in healthcare professionals. The present study aims to contribute to a better understanding of these relationships. Therefore, the following two hypotheses were formulated:

### Hypothesis 1

Higher perceived relationship quality between followers and leaders (LMX) reported from the followers’ perspective is associated with a lower perceived effort-reward ratio (ERI ratio).

### Hypothesis 2

Higher perceived importance of social relationships is associated with a greater impact on the interplay between the leadership-member exchange and effort-reward imbalance (ERI).

## Methods

### Study design and population

The data collection for this study was integrated into a broader research initiative and resulted in further investigations of several outcome variables, as well as differentiated subgroup and sub analyses of associations between transformational leadership, leader-member exchange and well-being, which have been published elsewhere [2, 9]. Therefore, there may be similarities in describing how the study proceeded and in describing the samples. This research includes a comprehensive, anonymous survey conducted in a large, representative tertiary hospital in Germany. The study was conducted prior to the onset of the COVID-19 pandemic and used a cross-sectional cohort design with a within-subject design. The survey was sent by e-mail invitation to a total of *N* = 10,101 leaders and followers from different professional groups. These included professionals with direct patient contact (doctors, nursing staff, therapeutic professionals) as well as those without direct patient contact (administration, IT, clinical services, office assistants, scientists).

### Ethics

The study was approved by the responsible ethics committee (No. 622/2017BO2), by the chief executive management board and the employees’ council of the tertiary hospital where the study was conducted.

### Measurements

An online survey via the Unipark survey software (QuestBack GmbH) was used to collect demographic information (age, gender, occupation, full- or part-time employment) as well as effort-reward imbalance (ERI), the dyadic relationship quality of leader and follower (LMX) and a rating of perceived importance of social relationships in the workplace with a polarity profile of stress dimensions.

### Effort-reward imbalance (ERI)

Effort-reward imbalance was measured using the German version of the Effort-Reward Imbalance questionnaire (ERI-S 10; [16, 54]) with two sub-scales (i) effort (consisting of three items, e.g. ‘I have constant time pressure due to a heavy workload’, ‘I have many interruptions and disturbances while performing my job’) and (ii) reward (consisting of seven items, e.g. ‘Considering all my efforts and achievements, I receive the respect and prestige I deserve at work’, ‘My job promotion prospects are poor’). Participants responded on a 4-point Likert scale ranging from 1 (strongly disagree) to 4 (strongly agree). Both scales show high internal consistency, with Cronbach’s alpha coefficients of *α* = 0.80 for effort and *α* = 0.84 for reward [55]. An effort-reward ratio = 1 (see Siegrist and colleagues [17]) indicates a balance between reported effort and reward. If individuals report less effort for each reward, the effort-reward ratio is < 1. In contrast, a ratio > 1 indicates that more effort is expended than reward received, indicating an imbalance.

### Quality of the leader-member-relationship (LMX)

To assess the quality of the leader-follower relationship (LMX [56]), we use the German version of the LMX questionnaire, which is based on the LMX model of Graen and Uhl-Bien [30] and consists of 7 items on a 5-point Likert scale. Participants are asked to rate seven questions and statements on a five-point Likert scale from 1 (low relationship quality) to 5 (high relationship quality), either in a version for leaders to rate the relationship quality with a prototype *follower (e.g. ‘How well do you understand your member`s job problems and needs’*,* ‘How would you characterize your working relationship with your member?’)* or in a version for followers to rate the relationship quality with their *direct* leader (e.g. *‘How well does your leader understand your job problems and needs?’*,* ‘How would you characterize your working relationship with your leader?’).* An average LMX score was calculated to assess relationship quality, Graen and Uhl-Bien [30] suggested that the LMX-7 assesses the three highly correlated relationship aspects of respect, trust and obligation as one LMX dimension (see Stuber et al. [2]). The LMX-7 has high internal consistency for employee ratings with Cronbach’s alpha coefficients of *α* = 0.89 and 0.92 [56].

### Importance of perceived social relationships (polarity profile)

The questionnaire developed by Michaelis et al. [45] and Junne et al. [42] measures the perceived importance of different areas of one’s own workplace in relation to individually experienced work-related stress with the technique of *polarity profile*. The questionnaire consists of four different dimensions with a total of 17 items and was developed based on an early German version of the Copenhagen Psychosocial Questionnaire (COPSOQ [57], for details see Michaelis et al. [45]). The dimensions are: ‘job demands’ (4 items), ‘influence and development opportunities at work’ (2 items), ‘working conditions’ (3 items) and the dimension relevant to this study: *‘social relationships and leadership’* (8 items in total: ‘social relationships at the workplace’; ‘communication culture at the team’; ‘organization culture at the team or the enterprise’; ‘management structure in the enterprise’; ‘style of leadership of superior’; ‘recognition and appreciation at the workplace’ and ‘perception of unfairness’).

Participants were asked to rate the perceived importance of the items with the question: *How important do you think the following aspects are for work-related stress at your workplace?* The response was given on a 6-point Likert scale from 1 (not at all important) to 6 (very important). A higher value indicates that the participant considers that aspect to be more important for work-related stress than items with a lower value.

### Statistical analysis and model construction

Means, standard deviations, frequencies and percentages were calculated to report sample characteristics. Percentage frequencies were calculated to analyse the effort-reward imbalance (ERI) relationship from the perspective of leaders and followers. In addition, *t*-tests were used to compare the perceived relationship between effort and rewards between leaders and followers, and Welch tests were used to compare the three largest occupational groups, specifically doctors, nurses and administration, from both perspectives. Comparisons of perceptions of relationship quality between leaders and followers were made using *t-*tests. Welch-tests were used to compare perceptions of relationship quality between each occupational group from the perspectives of followers and leaders.

In a sub-analysis to examine the relationship between leader-member exchange (LMX) and ERI and the effect of the importance of social relationships at work on this relationship, only data from participants who identified themselves as followers were combined as a sub-sample. Therefore, a multiple linear regression including moderation analysis was used to examine the relationship between LMX and ERI, as well as the moderating effect of perceived importance of social relationships at work. The assumptions of a multiple regression analysis were checked, Cohen’s *f*^2^ was reported as the effect size, with *f*^2^ ≤ 0.02 indicating a small effect, *f*^2^ ≤ 0.15 a medium effect and *f*^2^ ≤ 0.35 can be interpreted as a large effect size [58]. The LMX-7 sum score and the social relationship at work item score (both variables included in the product term of the interaction) were grand mean centered, as this allowed a more meaningful interpretation of the conditional effects of these variables and the intercept [59]. The level of significance for all analyses was α = 0.05. All calculations were carried out using IBM SPSS version 27 [60].

## Results

### Descriptive statistics

A total of *N* = 1,137 completed the survey (RR = 11.26%). Among the overall sample, *n* = 728 (70.5%) were female and *n* = 305 (29.5%) were male. The majority was older than 55 years (22.3%). 27.7% (*n* = 315) of the respondents considered themselves leaders and 72.3% (*n* = 822) followers. In addition, at the time of the survey, 57.4% (*n* = 462) of followers and 79.0% (*n* = 244) of leaders were working full-time. For more details on the sample, see Stuber et al. [2]. Table 1 reports on frequencies of occupations with and without patient contact and associated occupational groups divided by hierarchical level.

**Table 1:** Distribution of professional groups with and without patient contact by hierarchical level

Hierarchical level	Professional groups								
	With patient contact			Without patient contact					
	Doctors	Nursing staff	Therapeutic professionals	Administration	IT	Clinical service	Office assistants	Scientists	others
	*n*(%)	*n*(%)	*n*(%)	*n*(%)	*n*(%)	*n*(%)	*n*(%)	*n*(%)	*n*(%)
Followers	84(53.8)	142(67.6)	59(80.8)	157(70.4)	56(78.9)	8(72.7)	100(89.3)	87(77.0)	129(76.8)
Leaders	72(46.2)	68(32.4)	14(19.2)	66(29.6)	15(21.1)	3(27.3)	12(10.7)	26(23.0)	39(23.2)
Overall	156(13.7)	210(18.5)	73(6.4)	223(19.6)	71(6.2)	11(1.0)	112(9.9)	113(9.9)	168(14.8)

### Internal consistency

In the present study, internal consistencies were identified for effort-reward imbalance (ERI) for both followers and leaders and the exchange between leader and employees (leader-member exchange, LMX). When measuring ERI for *followers*, internal consistencies were found for the subscales effort (α = 0.77) and reward (α = 0.75). For *leaders*, internal consistencies were found for the subscales effort (α = 0.69) and reward (α = 0.80). For LMX, internal consistencies of α = 0.74 for leaders and α = 0.93 for employees were identified.

### Results for effort-reward-imbalance (ERI)

Leaders and followers differ significantly in their perceived efforts (range of reward scale 3–12) with *t*(834.71) = -12.11, *p* < .001. On average, leaders perceived more efforts with *M* = 10.75 (1.36) than followers with *M* = 9.45 (*SD* = 2.07). Both groups tend to be at the upper end of the scale. Leaders and followers differ significantly in their perceived rewards (range of reward scale 7–28) with *t*(1073) = -4.92, *p* < .001. On average, leaders perceive more rewards with *M* = 17.87 (*SD* = 4.1) than followers with *M* = 16.54 (*SD* = 3.9). Both, followers and leaders perceived more effort than rewards. Leaders and followers (overall and in the occupational groups) do not differ significantly in their perceived ratio (effort-reward-imbalance) with *t*(631.88) = -0.53, *p* = .15.

76.9% of followers and 88.1% of leaders reported a relative lack of rewards in relation to the level of effort (ERI). Significant differences were found between the occupational groups in the group of followers (Welch-test (2,229.32) = 17.97, *p* < .001). The post hoc tests showed that the occupational groups of doctors (ERI = 84.4%) and administrators (ERI = 69.7%) do not differ significantly in their perceived effort/reward ratio, whereas both occupational groups differ significantly from the occupational group of nurses (ERI = 91.8%) in their perceived effort/reward ratio.

### Results for leader-member exchange (LMX)

**Fig. 2:**
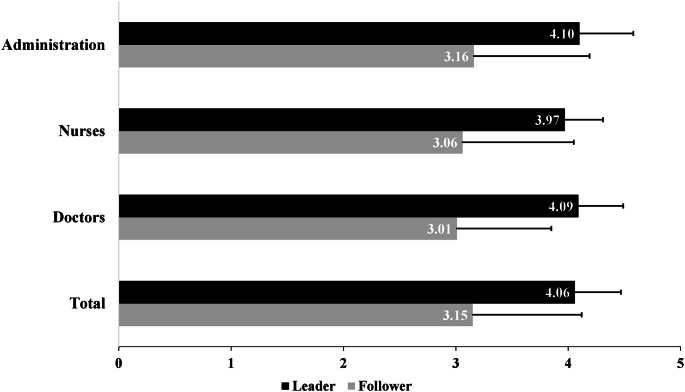
Mean values and standard deviations for the evaluation of relationship quality (LMX) from the perspective of leaders and followers of the total sample as well as divided by occupational groups

The results of relationship quality from the perspective of followers and leaders within the total sample and by professional group (doctors, nurses, administration) are shown in Fig. 2. Leaders rated the quality of the relationship between themselves and their followers as significantly higher than the reverse, *t*(1054,83) = -21.68, *p* < .001. This result is not only evident for the total sample, but also for the individual occupational groups. No significant difference in relationship quality was found between the three occupational groups, neither for followers nor for leaders.

### Moderated regression analysis

*General Result: N* = 650 of the followers were included in the regression analysis^1^. The total variance in effort-reward imbalance (ERI) explained by the model was 27.62%, *R*² = 0.28, *F*(6, 643) = 40.88, *p* < .001. This gives an effect size of *f*² = 0.38, which can be interpreted as a large effect [58]. All regression coefficients with corresponding standard error, test statistic, *p*-value (significant coefficients in bold) and confidence interval are listed in Table 2. The intercept indicates that the model predicts an effort-reward ratio of 1.35. Neither the control variable *follower gender*, *t*(649) = 0.56, *p* = .576, nor the control variable *leader gender*, *t*(649) = 0.91, *p* = .365, reached statistical significance. This indicates that neither control variable was significantly associated with ERI. The control variable profession group reached statistical significance, *t(*649) = -4.47, *p* < .001, indicating that a person working in a non-patient contact profession experienced an average of 0.20 units lower effort-reward ratio than a person working in a patient care profession.

The conditional effect of leader-member exchanges was significant (*t*(643) = -13.55, *p* < .001), indicating that leader-member relationship (LMX) was significantly associated with ERI. For participants with an average perceived importance of social relationships at work, their effort-reward Ratio was 0.28 units lower when their LMX increased by one unit. Thus, the higher the leader-member exchange, the lower the ERI. The conditional effect of individually perceived importance of social relationships at work also reached statistical significance (*t*(643) = 3.61, *p* < .001), meaning that perceived importance of social relationships was significantly related to ERI. For follower with an average of leader-member exchanges, the ERI increased by 0.065 when people rated the importance of social relationships one unit higher. The moderation was also statistically significant, *t*(643) = -2.30, *p* = .022 indicating that the relationship between the quality of leader-member exchanges and the effort-reward ratio was significantly moderated by the perceived importance of social relationships at work. Adding the moderation to the model explained an additional 0.59% of the variance in ERI, *F*(1, 643) = 5.28, *p* = .022, with an effect size of *f*² = 0.021, which can be interpreted as a small effect [58].

**Table 2:** Results of linear multiple regression analysis reporting effort-reward imbalance (ERI) as outcome: coefficients, standard errors, *t*-test value, *p*-value and confidence interval. Significant values are reported in bold

Model	β	SE(β)	t	*p*	CI_Lower_(β)	CI_Upper_(β)
Intercept	1.35	0.36	37.82	< 0.001	1.28	1.42
Follower gender	0.03	0.048	0.56	0.576	-0.07	0.12
Leader gender	0.04	0.042	0.91	0.365	-0.04	0.12
**Professional group** Followers with or without patient contact	-0.20	0.044	-4.47	< 0.001	-0.28	-0.11
**Leader-member exchanges (LMX)**	-0.28	0.021	-13.54	< 0.001	-0.33	-0.24
**Perceived importance of social relationships**	0.07	0.018	3.61	< 0.001	0.03	0.10
**Moderation**	-0.04	0.02	-2.30	0.022	-0.08	-0.01

#### Post-hoc analysis

**Fig. 3:**
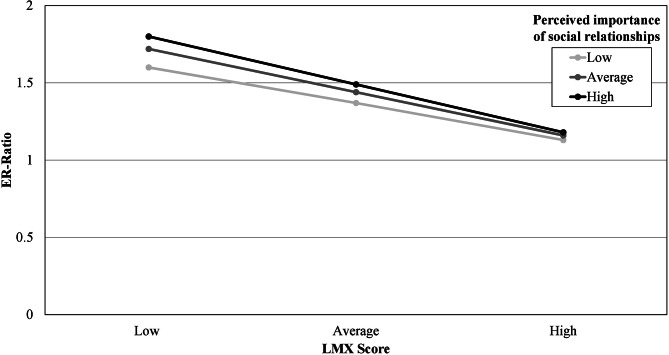
Association between effort-reward ratio (ER-Ratio) and leader-member exchange (LMX Score) for low, average and high perceived importance of social relationships

We conducted a post-hoc analysis to probe the interaction between LMX and three different levels (low, average, high) of the individual perceived importance of social relationships in the workplace regarding ERI with simple slopes. For conditioning *M* + 1 *SD* exceeded the maximum of the 6-point Likert Scale of importance of perceived social relationships. Therefore, we used the maximum scale score instead. For individuals with a low perceived importance of social relationships, each unit increase in LMX reduces the ERI by 0.24 units (LMX: *b* = − 0.24, *t*(643) = -7.68, *p* < .001). For people with an average perceived importance of social relationships in the workplace, each unit increase of LMX reduces ERI by 0.28 units (LMX: *b* = − 0.28, *t*(643) = -13.54, *p* < .001). For people who rated the importance of relationships in the workplace as high, an increase of unit regarding member-leader relationship reduced ERI by 0.32 units (LMX: *b* = − 0.32, *t*(643) = -12.98, *p* < .001). See Fig. 3 for an illustration of the simple slopes.

The Johnson-Neyman Technique shows a significant transition point at 3.56 units below the mean of the variable perceived importance of social relationships. This indicates that for scores less than two points there was no significant relationship between LMX and ERI. For scores of at least two points there was a significant relationship between LMX and ERI; *LMX b* = -0.14, *t*(643) = -1.96, *p* = .05. The relationship between LMX and ERI becomes more negative as the importance of social relationships increases. For the maximum score of `importance of perceived social relationships`, the relationship is negative with *LMX b* = -0.32, *t*(643) = -12.98, *p* < .001.

## Discussion

The present study investigated the quality of follower-leader relationships according to the leader-member exchange model (LMX) and the relationship with effort-reward-imbalance (ERI) among a group of healthcare professionals. Our main interest was to investigate the moderating role of perceived importance of social relationships at work on the relationship between LMX and ERI. It was expected that a higher perceived quality of the follower-leader relationship would be associated with a lower perceived effort-reward relationship from the employees’ perspective (hypothesis 1). Further it was expected that a higher perceived importance of social relationships would be associated with a greater impact on the interplay between the leadership-member exchange and effort-reward imbalance (hypothesis 2).

The results showed that more than 75% of employees reported ERI. Both leaders and followers experienced a relative lack of rewards in relation to the effort of their work. Those in direct patient care were most affected. In line with the first hypothesis, it was also found that a subjectively higher quality of the leader-follower relationship was associated with lower ERI. This relationship was moderated by the perceived importance of social relationships at work, consistent with the second hypothesis: The higher the subjective importance of social relationships, the stronger this relationship.

Overall, a high prevalence of ERI was shown among all employees in this study: the majority of employees (76.9%) and leader (88.1%) showed ERI, indicating above-average levels for ERI compared to the general working population [19]. The results for ERI were shown to be significantly higher than those in previous samples from the German healthcare system [61, 62] highlighting the ever-increasing pressure on the healthcare sector.

In addition, it was found that employees with patient contact experienced higher levels of ERI than those in less patient-facing professions. As previous studies have shown, patient contact itself is a stress-inducing factor [63, 64], which is further exacerbated by the social interaction between other healthcare professionals and emotionally distressed patients [65]. Accordingly, stress prevention and reduction are of particular interest to patient-facing professionals.

We found a conditional effect of LMX on the effort-reward relationship. Thus, high LMX quality appears to reduce the experience of ERI, highlighting the importance of the leader-follower relationship for employee health in the workplace hospital. These findings are consistent with previous research demonstrating the stress-preventing [38, 66] and health-promoting [1, 37] effects of LMX. This study also adds to previous findings on the positive impact of high-quality LMX on the occupational health system [67, 68]. Similar to the findings of Lankinen’s [39] study, we also found a relationship between LMX and the effort-reward ratio, with higher LMX quality reducing ERI. Another aspect that needs to be considered in the relationship between LMX and the imbalance between effort and reward is that the relationship between LMX and ERI is curvilinear rather than linear [52]: employees with low LMX, but also with particularly high LMX, experience a higher level of stress than employees with moderate LMX, which could be due to the increased expectations associated with high levels of LMX.

Furthermore, our findings are in line with previous studies, which have shown that social relationships at work can be considered an important potential ERI for employees [69–71]. We found that the importance of social relationships at work to have a conditional effect on ERI. That is, a person who perceived social relationships at work as important showed higher levels of ERI. In the same vein, Junne et al. [42] showed that social relationships are indicated as one of the most important stress-dimensions in work-settings from the differential views of human resource managers, occupational physicians, primary care physicians and psychotherapists.

Also, previous research has shown a stress-reducing effect of supporting social relationships at work on stress-related constructs such as burnout [43] and mental health [44]. Depending on the content of the support, this may even have the opposite effect and have a negative impact on the stress variables [72]. Accordingly, the importance of social relationships might be more present and important for people with very high or low levels of social support than for people with average levels of social support [73]. Also, as there is great heterogeneity in the use of instruments, the need for validated instruments to measure social support should be emphasized [74].

In addition, the present study found a moderating effect of the perceived importance of social relationships at work on the relationship between LMX and ERI. In this sample, higher perceived importance of social relationships was associated with a stronger impact of LMX on ERI. More specifically, the more important the follower-leader relationship, the more the quality of that relationship impact the stress experienced. As described earlier, social support itself at work has been shown to be a moderator of occupational stress [75, 76].

As LMX is a dyadic approach, employee characteristics have an impact on the quality of LMX [32]. For example, previous studies have highlighted the link between followers’ need for leadership, emotional intelligence and work performance with higher LMX quality [47–49]. Only a few studies have investigated the enhancement of LMX effects. For example, Dunegan et al. [77] found that high intrinsic employee satisfaction moderated and thus enhanced the effect of LMX on job performance in a hospital setting. Similarly, we found that high perceived importance of social relationships had an impact on the magnitude of the association between LMX and ERI.

### Strengths and limitations

This study demonstrates that the perceived importance of social relationships in the workplace hospital moderates the relationship between leader-member exchange (LMX) and effort-reward imbalance (ERI) in a large, representative tertiary hospital in Germany. To the best of our knowledge, this is a novel finding that has not yet been examined in any other study for the hospital workplace. A further advantage is that the results were obtained prior to the pandemic that occurred in 2019. This eliminates the possibility that pandemic-related stressors could influence stress at the workplace. For other studies examining the impact of the COVID-19 pandemic on health workers’ work stress and the influence of social support see e.g. [78, 79].

Despite the strength of the results, the study has some limitations. The design of the study was cross-sectional. This means that the relationships and models found should be interpreted in an associative rather than a solely causal way. The response rate was 11.26%. We suppose that the response rate would have been higher if was calculated based on the number of people who actually read the email, rather than the amount of email addresses. Also, the number of respondents who actually read the email would probably be higher, as not all addressees will have opened the email (see also Erschens et al., [9]).

The present study provides further evidence of the link between the perceived importance of relationships and their influence on stress experience. However, it is important to note that the present study did not measure direct social support or its quality. Therefore, the level of social support an employee receives could have influenced the perceived importance of this relationship.

It is also essential to highlight that within the regression model, the focus was solely on the perspective of followers and not that of leaders in relation to LMX. Sin et al. [50] showed that employee and leaders ratings were only moderately correlated.

In addition, further longitudinal studies can shed light on how the quality of relationships and the experience of stress between leaders and those they lead change over time, and how the perceived importance of social influences relationships between leaders and those they lead and the experience of stress at work over time.

It is essential to acknowledge that this study was conducted in a single tertiary university hospital in Germany. Consequently, the transferability of the results should be considered regarding the site-specific characteristics. Therefore, local, regional, and national contexts have to be taken into account when integrating the findings of this study into a national and international framework. For illustration, the protection and promotion of mental health in the workplace is of particular significance in Germany. In 2013, the Occupational Safety Act was expanded to include the employer’s duty of care, which subsequently encompassed the mental health of employees [80]. Moreover, specific organizational benefits and resources merit consideration. These include the presence of a company-wide health management system [81], human resources training and development, assistance in locating suitable accommodation, opportunities for achieving a healthy work-life balance (e.g. through an on-site clinic, daycare for children or sports programs for employees) as well as access to the company’s conflict management system.

Nevertheless, the multifaceted responsibilities inherent to a university hospital, including patient care, academic duties, research activities, and student education, can also give rise to elevated stress levels among employees.

Furthermore, context- and occupation-specific factors exert a considerable influence on the psychosocial well-being of healthcare workers. For instance, doctors in Germany report greater job satisfaction with their salaries than nurses [82]. Similar findings were observed with regard to job satisfaction among Finnish employees in university hospitals, with the group of doctors exhibiting the highest levels of satisfaction and the group of nurses demonstrating the lowest levels of job satisfaction [83]. Also, a large-scale study conducted in hospitals across various European countries. A significant discrepancy was observed in the perception of the psychosocial safety climate in teamwork between clinical management and frontline clinical staff in hospitals in several European countries, including Poland, Germany, France, and Spain [84].

Furthermore, the influence of health policy and the regulatory environment on organizational structure and resource availability can subsequently impact stress levels and the quality of communication between managers and staff. It must be acknowledged that the findings may not be generalizable to other international and EU-specific contexts.

Therefore, it can be posited that in other countries, alternative stressors and resources may exert a significant influence on the experience of distress among healthcare professionals. Consequently, as effective leader-member exchange (LMX) requires regular and open communication, time and resource constraints may have a negative impact on the quality of the relationship between followers and their leaders. To illustrate, a high patient-to-doctor ratio, as is common in countries such as Latvia, Romania, or Serbia, can have a regional impact on the stress levels experienced by healthcare professionals [85].

Moreover, cultural attitudes towards hierarchy and authority can influence the dynamics of follower-leader interactions. In more hierarchical cultures, there is a greater potential for less open communication and lower-quality exchanges, as hierarchical cultures often discourage the free flow of information and feedback [86, 87]. Finally, it is important to note that there are significantly fewer studies analyzing hospital staff in low and middle-income countries [88]. To ensure further external validity, future studies should be conducted on an international scale, allowing for the incorporation of international requirements for healthcare professionals.

### Implications and further directions

This study highlights the levels of stress in the hospital workplace and therefore the need for stress prevention measures. A significant proportion of the surveyed followers and leaders indicated that they perceived a discrepancy between the rewards they received for their efforts and the demands placed upon them in their current work environment. To offset this imbalance, there is the potential to either enhance the perceived reward or to diminish the perceived demands. In the professional context, reward is understood to encompass both material and immaterial gratification. The latter includes, appreciation and recognition, career opportunities and job security, prestige, status, and financial compensation. Perceived demand may refer to job-specific factors, including work and time pressure, workload, and the intrinsic willingness of employees to expend energy. Potential practical improvements in terms of rewards can be internally established, for example on team days. In doing so, the team can agree on which measures could lead to a change in the perception of the relationship between demands and rewards at the management level and in terms of cooperation between colleagues. Another method of enhancing the perceived reward is to implement regular, structured performance feedback from the manager. Additionally, a variable salary system based on requirements and performance can contribute to an increase in the perceived reward [89–91]. With regard to effort and reward, it should also be noted that job stress in the clinical setting can also have a positive effect on healthcare professionals’ satisfaction and commitment [92], underlining the importance of functional coping strategies [93, 94].

Our study provides evidence that leaders can have a significant impact on their followers’ experience of stress, highlighting the importance of training leaders in health-promoting leadership styles [2, 9, 95–97]. In line with Ruotsalainen et al. [98], we suggest that there is an urgent need for high-quality studies on stress prevention by offering comprehensive stress-reducing interventions to healthcare workers addressing structural as well as behavioural prevention. This would be an important step towards improving mental health and leader-member exchange (LMX) among all hierarchical levels and professional groups in the healthcare sector. Furthermore, the findings suggest that followers are significantly involved in shaping the relationship with their respective leader, which strengthens the perspective and individual influence of followers [99].

In sum, our findings shed light on the importance of social relationships in the workplace and the need for stress prevention measures for both leaders and, in particular, healthcare workers. In addition, the need for specific training measures aimed at strengthening positive interactions and thus relationships in the workplace is apparent. This study provides further evidence that the perceived importance of social relationships at work also has an impact on LMX. Overall, this new finding contributes to the current understanding of LMX theory and highlights that follower characteristics can influence the level of LMX, emphasizing that the follower perspective should also be included and considered relevant.

## Data Availability

There are legal restrictions on sharing this deidentified data set. Due to the high confidentiality of the data, the authors of the study received permission from the Medical Faculty of the hospital, specifically the Chief Executive Board of the Hospital and the Employees’ Council of this hospital to conduct the study and to collect these data only if they were not made publicly available without individual permission for specific questions (i.e., upon request). Data requests may be sent to Prof. Dr. med. Stephan Zipfel, Medical University Hospital Tuebingen, Osianderstr. 5, 72076 Tuebingen/Germany, (stephan.zipfel@med.uni-tuebingen.de).

## Footnotes

1 Due to incomplete data, 172 participants were excluded.
